# Linked alterations in structure and autoimmunity biomarkers in remote mild-to-moderate TBI: A multi-modal brain imaging study^☆^

**DOI:** 10.1016/j.nicl.2025.103838

**Published:** 2025-06-30

**Authors:** Abigail B. Waters, Samantha H. Penhale, Shoumi Sarkar, Somnath Datta, Damon G. Lamb, Claudia Robertson, Richard Rubenstein, Amy K. Wagner, Firas Kobeissy, Kevin Wang, John B. Williamson

**Affiliations:** a Brain Rehabilitation Research Center, North Florida/South Georgia VAMC, Gainesville, FL, USA; b Department of Clinical and Health Psychology, University of Florida, Gainesville, FL, USA; c Department of Biostatistics, University of Florida, Gainesville, FL, USA; d Department of Psychiatry, Center for OCD and Anxiety Related Disorders, University of Florida, Gainesville, FL, USA; e Department of Neurosurgery, Baylor College of Medicine, Houston, TX, USA; f Department of Neurology, SUNY Downstate Health Sciences University, Brooklyn, NY, USA; g Department of Physical Medicine and Rehabilitation, University of Pittsburgh, Pittsburgh, PA, USA; h Department of Neurobiology, Center for Neurotrauma, Multiomics & Biomarkers (CNMB), Neuroscience Institute, Morehouse School of Medicine, Atlanta, GA, USA

**Keywords:** Traumatic brain injury, CNS biomarkers, Multimodality neuroimaging

## Abstract

**Background:**

There is significant heterogeneity in the recovery course from mild-to-moderate traumatic brain injury (mmTBI), with many individuals reporting cognitive symptoms during the chronic phase. Although blood-based biomarkers have been identified as a marker of injury severity in the acute phase, the relevance of candidate biomarkers in chronic mmTBI is less clear. Establishing links between blood-based biomarkers, neuroimaging, and cognitive performance is necessary to differentiate subphenotypes in chronic TBI and improve prognostic models.

**Methods:**

Sixty Veterans and non-Veterans with mmTBI completed cognitive testing (WAIS-IV), MRI, and blood collection for blood-based CNS biomarker assessment for cross-sectional comparison. A data fusion technique (Linked Independent Component Analysis [LICA]) was used to simultaneously model structural variability across T1-weighted and diffusion-weighted MRI modalities. Blood serum samples were assayed using an ultrasensitive immunoassay using digital array technology to measure GFAP, NFL, total tau, and UCH-L1 protein levels. A correlation matrix was used to identify which LICA-derived MRI components were associated with (1) a blood-based biomarker, (2) a WAIS-IV index score and (3) clinical characteristics of TBI, using an effect-size cut-off.

**Results:**

LICA-derived Component 4 was associated with a biomarker (UCH-L1), reduced processing speed, and the total number of TBIs. This component was characterized by increased mean diffusivity along the ventral surface of the frontal lobe and decreased fractional anisotropy in bilateral corticospinal tracts and cerebral peduncles.

**Conclusions:**

Our findings contribute to the larger body of literature examining the utility of biomarkers for chronic TBI and underscore the importance of examining heterogeneity within this population.

## Highlights

• This is the first study of chronic TBI using linked independent component analysis.
• Diffusion alterations were associated with TBI history, in chronic TBI.
• Linked structural variability in the brain was associated with cognition and UCHL1.

## 1 Introduction

There is significant heterogeneity in the initial clinical presentation, recovery course, and symptom sub-phenotypes within mild-to-moderate traumatic brain injury (mmTBI), with a substantial portion of individuals reporting persistent symptoms after the expected recovery timeframe (Cancelliere, 2023). This heterogeneity impedes the identification of at-risk individuals, communication of prognosis to patients, and the development of effective therapeutic interventions for cognitive symptoms. The validation of blood-based biomarkers for diagnosis and prognosis is critical to help differentiate subphenotypes in TBI and to identify potential treatment targets, particularly for injuries that are less severe without obvious primary injury on clinical imaging.

Sensitive assay techniques have identified candidate blood-based biomarkers that reflect the pathophysiological processes related to TBI (e.g., neuronal/axonal/glial cell injury, disruptions of the blood–brain barrier, neuroinflammation), particularly in the acute phase (Zetterberg and Blennow, 2016). Identified candidates include ubiquitin C-terminal hydrolase-L1 (UCH-L1) as a marker of neuronal injury, neurofilament light (NF-L) and tau as markers of myelinated and unmyelinated axonal injury respectively, and glial fibrillary acidic protein (GFAP) as a marker of astroglial injury (Zetterberg and Blennow, 2016). Acutely, elevations in UCH-L1, NF-L, GFAP, and tau have been observed in mild and moderate TBI that are continuously associated with indicators of more severe injury(e.g., loss of consciousness, intracranial injury (Kulbe and Geddes, 2016, Papa, 2012, Papa, 2016). However, some of these same biomarkers (GFAP, NF-L) are not significantly associated with post-concussive symptoms in the subacute recovery period (Clarke, 2024, Boucher, 2023) (<3 months post-injury). Therefore, the relevance of these biomarkers to chronic mmTBI remains unclear, both in characterizing the impact of the original injury and in identifying vulnerability that persists over time. Additional research is needed to explore their relationship to cumulative TBI burden in chronic populations and determine their value to prognostic modelling of brain aging.

To validate biomarkers and increase clinical utility, it is important to establish links between biomarkers and important functional outcomes. In addition to somatic and psychological symptoms (Cancelliere, 2023), subjective cognitive complaints are also common among individuals with persistent symptoms following mmTBI (Levy et al., 2023), especially for older adults (Peters, 2016) and those with increased injury burden (e.g., loss of consciousness, multiple injuries; Lennon, 2023). Tests assessing processing speed and working memory are particularly sensitive to mmTBI (Carlozzi et al., 2015). However, these findings are not specific to TBI and weaknesses in working memory and processing speed can also be associated with common co-morbid conditions such as depression (Terry et al., 2019), PTSD (Thompson et al., 2024), and poor sleep (Anderson and Jordan, 2021). Identifying convergence between multiple modalities of evidence (e.g., neuroimaging, blood-based CNS biomarkers, cognitive testing) will help to disentangle the unique contributions of mmTBI. Given the diffuse distribution of candidate biomarkers in the brain, and their potential as a marker of vulnerability to cognitive aging, the integration of neuroimaging from multiple modalities may provide a more complete understanding of the relationship between blood-based CNS biomarkers and brain structure. Furthermore, the joint analysis of imaging data is better suited for exploring complex relationships (Calhoun and Sui, 2016) in chronic mmTBI, where the signal-to-noise ratio may be weaker.

Linked Independent Component Analysis (LICA) is a Bayesian extension of independent component analysis which has been used as a data-fusion technique for multi-modality neuroimaging, without spatial concatenation across modalities which is markedly impacted by scaling (Groves et al., 2011, Groves, 2012). By identifying convergence of evidence across multiple modalities, LICA analyses increase statistical power with robustness to noise and ease interpretation by assuming regions co-vary together (Calhoun and Sui, 2016). In samples of adults without TBI, LICA has been shown to reliably identify large-scale multi-modal networks associated with cognitive aging, known cardiovascular risk factors, and vulnerability to neurodegenerative disease (Groves, 2012, Douaud, 2014, Kharabian Masouleh, 2018). Examining the relationships between these multi-modal components and blood-based biomarkers (e.g., UCH-L1, NF-L, GFAP, tau) is an under-explored area with significant potential to inform complex post-injury mechanisms in TBI.

A single study has examined linked MRI components (diffusion MRI and resting state functional connectivity) in recovery from TBI (Manning, 2019). In a sample of 21 female athletes with concussion, repeat imaging within the acute (24–72 h post-injury) and subacute (3 months and 6 months post-injury) recovery period, found that acute changes in diffusion metrics within deep white matter tracts, including the corticospinal tract, were linked to variability in resting-state functional connectivity across large-scale cognitive networks. Long-term alterations of brain structure at 6 months were observed along the body and splenium of the corpus callosum, fornix, and thalamus, which were linked with hyperconnectivity of the default mode network, executive control network, and cerebellar network; this component was also associated with the total number of concussions reported. Notably, analyses revealed significantly more variability in functional connectivity and a predominance of structural features within meaningful components. Although this study underscores the potential of data fusion techniques to leverage multi-modality neuroimaging in mmTBI, there remain significant gaps in the literature in the understanding of heterogeneity of different outcomes.

The primary aim of this study was to characterize structural variability in the brain associated with the cognitive symptoms, clinical characteristics, and blood-based biomarkers of neuronal, axonal, and astroglial injury (i.e., GFAP, NF-L, UCH-L1, and tau) in mmTBI. To our knowledge, ours is the first study to utilize LICA to examine linked components in chronic mmTBI. In addition to the difference in time period, we expanded on the previous investigation (Manning, 2019) by incorporating measures of gray matter morphology to gain a richer understanding of the structural variability in mmTBI, which appeared to be more consistent, in contrast to functional connectivity.

## 2 Methods

### 2.1 Participants and procedures

Seventy-five Veterans and 19 non-Veterans with remote mmTBI were recruited from the North Florida/South Georgia Department of Veterans Affairs Medical Center and surrounding community. Seventy-two individuals, after inclusion/exclusion evaluation (see below) completed remaining study procedures across two visits. Twelve participants were excluded from statistical analyses due to incomplete neuroimaging and blood-serum data. The final sample consisted of 60 participants. All study procedures were approved by the University of Florida Institutional Review Board. Participants provided written informed consent and were compensated for their time and travel.

Joint VA and DOD diagnostic guidelines for TBI were used for this study. TBI was defined as any injury to the head as a result of blunt trauma or blast injury with any period of (observed or self-reported): transient confusion, disorientation, impaired consciousness, dysfunction of memory immediately before or after the time of injury, and/or signs of neurological or neuropsychological dysfunction identified soon after injury (within minutes). Individuals who reported less than 30 min of loss of consciousness (LOC) were classified as having a mild TBI; those who reported between 30 min and 24 h LOC and/or primary injuries to the brain (e.g., bleeding on acute imaging) were classified as having a moderate TBI. Individuals who reported a mild or moderate TBI at least 6 months prior to study enrollment were eligible for participation.

Eligible individuals underwent a screening visit where basic demographic information (including service details as applicable), prior TBI experience, medical history, psychiatric history (including PCL-5), and substance use history were collected via structured interview, including the OSU-TBI identification method (Corrigan and Bogner, 2007) to characterize TBI history and clinical characteristics. Additionally, cognitive screening was completed consisting of the Montreal Cognitive Assessment (MoCA) and a performance validity measure (Test of Memory Malingering; TOMM). Exclusion criteria for the study included: history of severe TBI with LOC greater than 24 h, major medical illness (including non-TBI related neurological disease), major uncorrected sensory deficit, current drug or alcohol abuse, history of severe psychiatric illness (e.g., schizophrenia, bipolar disorder), pregnancy, any contraindications for MRI scanning (e.g., metal fragments, incompatible implants), a MoCA score of ≤ 18, and/or failure of the performance validity measure at the screening visit (TOMM Trial 2 < 45). Eligible participants completed a half-day study visit, which included collection of cognitive testing, blood collection and processing for blood-based CNS biomarker assessment, and MRI.

### 2.2 TBI burden

The lifetime burden of TBI was tested two ways: total number of TBIs (capped at 5) and a derived TBI burden score. The derived TBI Burden score was calculated for each participant based on self-reported history of TBI, including total number of TBIs, instances of LOC, and instances of alteration of consciousness only (AOC; i.e., memory gaps, confusion). Each clinical characteristic was treated as ordinal and capped at 5, to address skew in the sample. A principal component analysis (PCA) was used to determine the best linear combination of TBI characteristics which explained the greatest amount of between-subject variance in TBI history. For each PCA-derived component, each instance of TBI, AOC, and LOC is assigned a weight, and the sum of the assigned weights is used to calculate the total amount of variance explained. The component which explained the most variance (50.7 %) was used to calculate individual TBI burden scores. See Supplemental Table S1 for the relative weights of TBI-instance characteristics for the TBI Burden score.

### 2.3 Cognitive testing

Participants completed a full neuropsychological battery, which included select subtests from the WAIS-IV (Digit Span, Letter-Number Sequencing, Digit Symbol, and Coding). Two index summary scores were included in analyses: the working memory index (WMI; derived from Digit Span and Letter-Number Sequencing subtests) and the processing speed index (PSI; derived from Digit Symbol and Coding subtests). Index scores were calculated using published, age-corrected norms.

### 2.4 Measurement of blood-based CNS biomarkers

Blood serum samples were assayed using an ultrasensitive immunoassay using digital array technology (Quanterix Single Molecule Arrays [SIMOA]-based Human Neurology 4-plex B assay [N4PB]) to measure GFAP, NFL, total tau, and UCH-L1 protein levels in pg/mL.

### 2.5 Individual modality pre-processing

All imaging was conducted at a scanning facility located at the University of Florida, using a 3 T Siemens MRI scanner. T1-weighted anatomical scans (TE/TR = 2.26/1800 ms, 1.0 mm^3^) and diffusion-weighted MRI (dMRI) were collected for each participant (TE/TR = 89/4000 ms, 2.0 mm^3^, b ≤ 3000, 180 directions). Cortical reconstruction and volumetric segmentation were performed in Freesurfer (Fischl, 2012) on T1-weighted images to extract cortical thickness (CT) and pial area (PA). Additionally, voxel-based morphometry (VBM) was generated using the computational analysis toolbox (CAT-12) for SPM12 (Ashburner and Friston, 2005). CT, PA, and VBM were down-sampled to 2 mm isotropic and smoothed with a 10 mm FWHM Gaussian smoothing kernel, for computational efficiency (Groves et al., 2011, Groves, 2012).

DMRI was corrected for subject motion, eddy current distortion, and susceptibility-induced distortion, in FSL (6.0.6) with reverse phase-encoded field maps. The neighboring DWI correlation, or the voxel-wise correlation coefficients of low-b diffusion volumes, was calculated for quality control. DMRI data was then reconstructed into MNI space using q-space diffeomorphic reconstruction (QSDR) with a sampling ratio of 1.7 (Yeh and Tseng, 2011), and co-registered with each participant’s T1-weighted anatomical scan in DSI-Studio (v.20220803). Voxel-wise normalized fractional anisotropy (FA) and mean diffusivity (MD) were extracted. FA images were thresholded at 0.2 and skeletonised using the FSL TBSS pipeline (Smith, 2006) for computational efficiency; MD images were thresholded at 0.2, consistent with prior literature (Groves et al., 2011, Groves, 2012).

### 2.6 Linked Independent Component Analysis (LICA)

**Fig. 1:**
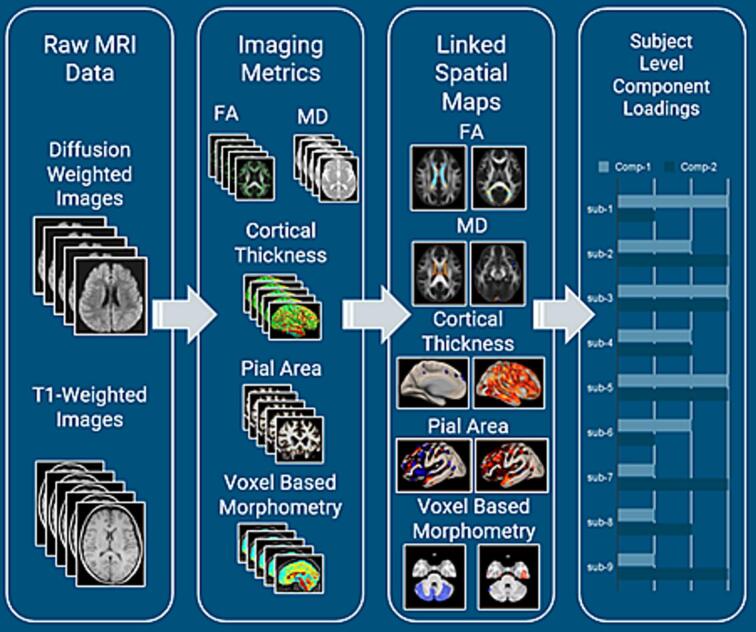
Linked Independent Component Analysis (LICA) pipeline.

Linked Independent Component Analysis (LICA) was used to simultaneously model structural variability across T1-weighted and diffusion-weighted MRI modalities (Groves, 2012) in FSL. Using LICA, data was decomposed into independent components across 5000 iterations (See Fig. 1). For each component, spatial maps for each imaging modality were extracted, displaying specific regions that co-vary across subjects, and subject-level component loadings were calculated.

The number of components, or model order, is selected a-priori, and generally limited by sample size (*N*/4). To avoid over-fitting, iterative model order was tested (*k* = 3–15) to confirm robustness of results (Wolfers, 2017). For each dimensionality the between-subject noise variability (StandardDeviation_Max_ < 2*StandardDeviation_Min_) and single subject dominance (α < 0.05) were evaluated for each component, as well as the correlation between the 15-dimensional factorization and the final model order selected (Llera et al., 2019).

### 2.7 Statistical analyses

A correlation matrix was used to identify which LICA-derived MRI components were associated with (1) a blood-based biomarker, (2) a WAIS-IV index score and (3) TBI burden score, using an effect-size cut-off (medium, *Cohen’s d* > 0.5). An exploratory analysis was also conducted with the presence of at least one moderate TBI as the marker of TBI burden (*N* = 6), using a Kruskal-Wallis test. Multiple linear regression was then used to determine if the relationships between subject-level LICA-component loadings, blood-based biomarkers, cognition, and TBI burden were independent, with biomarkers added on the first step, cognition added on the second step, and TBI burden on the third step.

## 3 Results

The final sample was predominantly White (80.0 %) and male (71.7 %), and approximately a quarter identified as Latino or Hispanic (26.7 %). Age was normally distributed, ranging from 19 to 59 (*M* = 36.55, *SD* = 10.92). All but one participant had at least 12 years of education, with 80 % holding at least an associate’s degree (61.9 %). More than ¾ of the sample were Veterans (See Table 1).

**Table 1:** Basic demographics and descriptive statistics.

	Total (*N* = 60)	
	*M (SD)*	*N (%)*
Age (years)	36.55 (10.29)	
Sex (Male)		43 (71.7)
Education (years)	14.60 (2.32)	
Race		
*White*		48 (80)
*Black*		6 (10)
*Biracial/Other*		6 (10)
Ethnicity (Hispanic/Latino)		16 (26.7)
Veterans		49 (81.7)
TBI Burden Score	1.25 (0.95)	
Months Since Most Recent TBI	107.71 (84.72)	
Number of TBIs	2.68 (1.93)	
*1*		17 (28.3)
*2*		15 (25.0)
*3*		18 (30.0)
*4*		3 (5.0)
*5 or more*		7 (11.7)
Number of TBIs with LOC	1.15 (1.33)	
*1*		19 (31.7)
*2*		14 (23.3)
*3*		2 (3.3)
*4*		1 (1.7)
*5 or more*		2 (3.3)
Number of TBIs with AOC	1.53 (1.33)	
*1*		19 (31.7)
*2*		14 (23.3)
*3*		8 (13.3)
*4*		3 (5.0)
*5 or more*		2 (3.3)
At least one moderate TBI		6 (10.0)
Biomarkers (pg/mL)		
*GFAP*	85.98 (104.91)	
*NFL*	7.58 (3.51)	
*UCH-L1*	11.85 (11.25)	
*Tau*	0.53 (0.78)	
WAIS-IV PSI	100.12 (12.33)	
WAIS-IV WMI	99.21 (13.58)	

Regarding TBI-clinical characteristics, 6 individuals reported a history of at least one moderate TBI and more than half (71.7 %) of the sample reported a history of multiple TBIs (≥2). Approximately half of the sample experienced at least one TBI with some period of LOC (63.3 %). Months since most recent TBI ranged from 12 to 336 months.

**Fig. 2:**
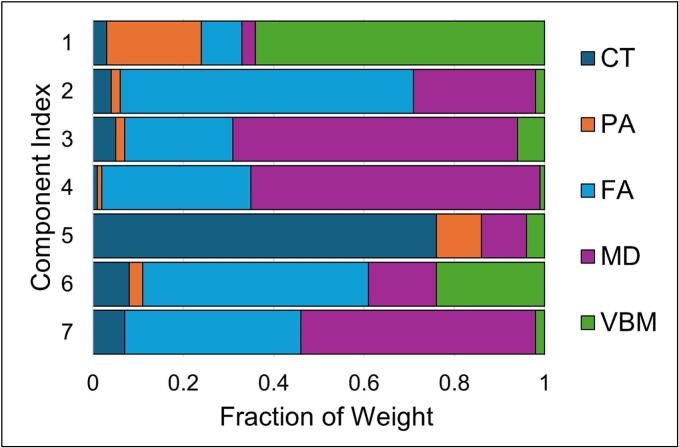
Proportion of each component that is accounted for by each modality. Cortical thickness (CT); Pial area (PA); Fractional anisotropy (FA); Mean diffusivity (MD); Voxel based morphometry (VBM).

Using LICA, 7 components were extracted with adequate between-subject noise variability (StandardDeviation_Max_ < 2*StandardDeviation_Min_) and without significant single subject dominance (α < 0.05). When model order was increased to 15, 6/15 components were dominated by single-subject contributions. Correlations between the corresponding components in the 15-dimensionality factorization demonstrated significant association (*r* > 0.95) with the 5/7 components extracted with the final model order, suggesting high reliability. The remaining 2 components (Component 5 and Component 7) in the 7-dimnesionality appeared to be split across two components each in the 15-dimensionality factorization, with correlations ranging from 0.43 to 0.80. The relative contribution from each imaging modality in the final model is presented in Fig. 2. Descriptions of each component are available in the Supplement. There were no significant differences for subject-level loadings between Veterans and non-Veterans on any component.

Of the 7 LICA-derived components, only Component 4 was associated with a biomarker (UCH-L1), cognitive test score (PSI), and the total number of TBIs (See Table 2). Component 4 was also associated with total TBI Burden, but this fell below the cut-off criteria (*Cohen’s d* > 0.5). However, Component 6 was associated with NFL and WMI; exploratory analyses revealed that Component 6 was also associated with history of at least one moderate TBI with a medium effect size (Kruskal-Wallis X^2^ = 7.00, *p* = 0.008, *n^2^* = 0.12). This appeared to be driven by a single, female subject (LICA-derived Component 6 loading = -3.40). The strong relationship between this subject and Component 6 may be explained by the VBM-contribution localized largely to the cerebellum, due to sex differences in cerebellar density (Steele and Chakravarty, 2018). When the outlier subject was removed, only WMI remained associated with Component 6 (*r* = 0.34).

**Table 2:** Correlation coefficients (*r*) between LICA-Derived Components (1–7) and domains of interest (biomarkers, cognitive functioning, and TBI burden). Correlations which met cut-off criteria (*Cohen’s d* > 0.5) are bolded.

	Components						
	**1**	**2**	**3**	**4**	**5**	**6**	**7**
Biomarker							
*GFAP*	0.07	**0.25**	−0.08	0.05	−0.03	−0.14	−0.02
*NFL*	−0.01	0.22	0.14	−0.17	−0.15	**−0.29**	0.14
*UCH-L1*	0.05	0.02	−0.07	**0.35**	0.11	−0.08	−0.05
*Tau*	0.10	−0.04	0.08	−0.04	0.02	−0.14	0.22
Cognitive							
*WAIS-IV PSI*	0.16	−0.16	0.14	**−0.26**	0.21	0.23	−0.24
*WAIS-IV WMI*	**0.28**	−0.06	0.01	−0.19	**0.27**	**0.31**	−0.16
TBI							
*TBI Burden*	0.09	0.02	0.06	0.23	−0.05	−0.01	0.01
*Total # of TBI*	0.08	−0.04	−0.01	**0.29**	−0.08	0.03	−0.01

UCH-L1 was independently associated with Component 4 (See Table 3). There appeared to be shared variance between total number of TBI and PSI, such that when the total number of TBI is entered on the third step, PSI is no longer significantly associated with Component 4. Although PSI was not significantly correlated with total number of TBIs (*r* = -0.15, *p* = 0.241) or TBI Burden score (*r* = -0.13, *p* = 0.335), the PSI was associated with history of TBI with LOC (*F* = 4.19, *p* = 0.045, *n^2^* = 0.07), even after controlling for PTSD symptoms (performed due to known association of PTSD to cognitive performance).

**Table 3:** Results of multiple linear regression predicting identified components with biomarker (UCH-L1) entered on the first step, cognition (WAIS-IV PSI) on the second step, and TBI Burden on the third step. Total *R^2^* is presented for the full model and the semi-partial (Δ*R^2^*) is presented for each predictor.

	Step 1		Step 2		Step 3		*Final*
	*B*	*p*	*B*	*p*	*B*	*p*	*R^2^*
**Component 4**							
UCH-L1	0.03	0.008	0.03	0.008	0.03	0.011	0.10
WAIS PSI			−0.02	0.045	−0.02	0.084	0.15
Total # of TBI					0.19	0.048	0.20

**Fig. 3:**
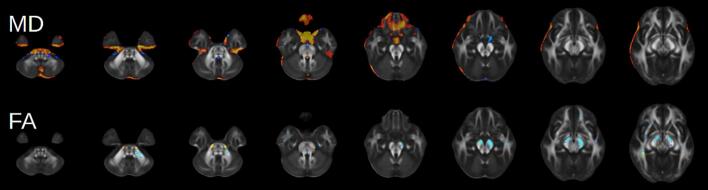
Visualization of LICA-derived Component 4.

Regarding multi-modality structural characteristics, the bulk of the variance in LICA-derived Component 4 was accounted for by MD (63 %) and FA (33 %). Visualized in Fig. 3, this component was characterized by increased MD along the ventral surface of the frontal lobe and decreased FA in bilateral corticospinal tract and cerebral peduncle. The contribution of MD is potentially suggestive of atrophy but may also represent some degree of microstructural change in grey matter that is not captured by CT, PA, and VBM.

## 4 Discussion

This study identified a convergence of evidence between multi-modal structural characteristics of the brain, aspects of cognition associated with TBI, clinical characteristics of cumulative TBI burden (i.e., total number of TBIs), and an identified candidate biomarker of chronic TBI. Associations with processing speed, the most consistent cognitive weakness in mmTBI, were found in regions of the brain vulnerable to TBI, across the ventral surface of the frontal lobe. Findings contribute to the growing body of literature investigating the use of biomarkers in chronic TBI and underscore the importance of examining heterogeneity in this population.

Primary associations between biomarkers, cognition, and number of TBIs were found across the ventral surface of the frontal lobe and motor tracts. Impact acceleration models in animals have found that the ventral regions of the brain are most vulnerable to changes post-injury, likely related to the contrecoup impact (Kikinis, 2017). This has been supported by case-control studies in humans with mmTBI (Eierud, 2014) and consistent with our own findings, demonstrating an association with ventral brain regions and lifetime TBI history. Furthermore, the LICA-derived component identified in this study shares many similarities with the multi-modality component associated with a possible inflammatory response in acute concussion (Manning, 2019). Although these effects appeared to resolve in athletes during the off-season, it is possible that repeated injuries may have more long-lasting effects from both initial injury characteristics and repeated neuroinflammation. Regional patterns of vulnerability to neuroinflammatory processes in the context of TBI may improve our understanding of these interactions.

Total number of TBIs was more strongly associated with the multi-modality imaging component, compared to the calculated TBI burden score, which weighted different instances of TBI, LOC, and/or AOC. Compared to the linear, numeric measure of total number of TBIs, the calculated TBI burden score weighted the second instance of TBI most heavily, as it explained the most variance across injury characteristics. This may suggest that the structural vulnerabilities identified may be more closely associated with a higher number of repetitive injuries. However, this difference should not be over-interpreted, as the association between the LICA-derived component and either measure of TBI burden trended in the same direction. Although only one meets criteria for statistical significance, there is not a significant difference *between* the strength of the associations.

Among individuals with mmTBI, reduced white matter integrity in these regions has also been associated with reduced motor functioning, including postural control (Zampieri, 2023), vestibular symptoms (Delano-Wood, 2015), and psychomotor processing speed (Muller, 2021), consistent with our findings. However, the relationship between processing speed and mild TBI, as a condition, has been more mixed in the literature, due to the confounding effects of comorbid conditions such depression (Terry et al., 2019), PTSD (Thompson et al., 2024), and sleep dysregulation (Anderson and Jordan, 2021), which may be in part contributed to by TBI, and likely heterogeneity in recovery. The partially shared variance between total number of TBIs and processing speed in our sample support the conclusion that cognitive deficits reported in mild TBI reflect the interaction between premorbid, injury-related, and perpetuating behavioral health and psychiatric factors.

As a marker of neuronal loss (Mondello, 2016), serum elevations in UCH-L1 have been found across the spectrum of severity in acute TBI, associated with poorer prognosis, and, in mmTBI, are most closely associated with primary intracranial injuries, headache, poor sleep, and cognitive symptoms (Papa, 2012, Papa, 2016, Diaz-Arrastia, 2014). Even in the subacute phase, UCH-L1 has been predictive of increased neurobehavioral symptoms during the chronic phase (Lange et al., 2023). However, the use of UCH-L1 in the chronic recovery phase is an understudied area of investigation. In mixed samples of chronic TBI, UCH-L1 has been associated with cognitive performance, although this relationship seems to be driven largely by more significant injuries (Lippa et al., 2021). A more recent study in chronic, mild TBI demonstrated reductions in UCH-L1 following neurorehabilitation that was associated with decreased post-concussive symptoms (Eagle, 2023). The findings from this study contribute to the larger body of research, demonstrating a relationship between UCH-L1 and cumulative TBI burden in a sample of chronic mmTBI. However, it is important to note that UCH-L1 levels observed in this sample largely did not meet proposed diagnostic cut-off criteria in acute TBI (Papa, 2012, Welch, 2016), which are sensitive but not specific to primary injury. Further research is needed to disentangle the degree to which biomarkers reflect primary injuries and/or chronic, cascading effects of TBI and/or associated co-morbidities.

Outside the context of acquired brain injuries, UCH-L1 may play a unique role in maintaining corticospinal motor neurons (Jara, 2015). In rodent models without UCH-L1 function, there is selective degeneration of corticospinal motor neurons, without impact on other motor projection neurons. This process is mediated through endoplasmic reticulum stress, which leads to the accumulation of unfolded proteins, and is linked to the metabolic cascade following TBI (Yang, 2024). Further research into the unique role of UCH-L1 and endoplasmic reticulum stress in TBI may identify targets for intervention and increase our understanding of UCH-L1 in chronic mmTBI.

Although we do not want to over-interpret the effects which did not meet our cut-off criteria, there are some notable patterns. First, total tau is the only biomarker that was not strongly associated with any LICA-derived brain component. This underscores the importance of examining tau-subspecies (e.g., p-tau 231), that may be more relevant to TBI. Second, while the WAIS-IV PSI and WMI were strongly associated with multiple LICA-derived components with a large range of modality predominance (e.g., WMI related to both a component dominated by cortical thickness and a component dominated by white matter metrics), biomarkers appeared to be more specific. This is not unexpected, given the multiple demands placed by any one neuropsychological test and reinforces their use as a relatively gross measure for regional brain functioning. Finally, the relationship between NFL and Component 6 appeared to be driven by a single female subject, which raises the question of sex-specific mechanisms. Given the sample distribution of this study, we are unable to fully explore the impact of sex, but this remains an underexplored and important area of research. Further, TBI heterogeneity and other causes of cerebellar volume differences cannot be ruled out.

There are several limitations to this study that represent important avenues for future research. This study used a cross-sectional research design in a sample of individuals with variable, remote history of TBI and thus our ability to determine causality is limited. This is further complicated by the frequency of confounding neuropsychiatric and medical conditions in Veteran populations, which contribute to cognitive deficits, although individuals in this sample were not considered cognitively impaired. Further, there is a complex relationship between neuropsychiatric conditions and TBI, such that TBI may be causally linked to their manifestation (Williamson et al., 2013). These conditions may perpetuate secondary injury (Wilson, 2019, Williamson et al., 2015) and the use of inflammatory biomarkers in these investigations may be particularly helpful. Although LICA has a number of advantages as a data-driven method to identify multi-modality patterns, there are a number of analytic decisions (e.g., number of modalities, model order) that can impact the component generation. In particular, the prominence of diffusion metrics may be attributable to differences in variance between modalities, which is not uncommon across studies using LICA and is improved compared to other methods of data fusion (Groves, 2012). Furthermore, it is not uncommon for imaging measures collected from one modality to co-vary more closely than those from another, which may explain the limited contribution from CT, PA, and VBM for Component 4, despite the evidence of possible atrophy and/or microstructural changes in MD. Although the consistency of our findings with the single prior LICA investigation (Manning, 2019) in TBI raise confidence in our findings, variability in data-driven imaging outcomes given the heterogeneity within chronic TBI samples merit further investigation. Finally, although we attempted to reduce the possibility of a Type I error with an effect size cut-off and by identifying convergence of evidence across imaging, behavior, and biomarkers, we did not use a correction for multiple comparisons for *p*-values, given the exploratory nature of this work.

In conclusion, using data-fusion techniques, we found associations between examine multi-modality structural characteristics in the brain, TBI-related serum biomarkers, cognitive performance, and total number of TBIs. This represents the first study to utilize LICA to examine linked components in chronic mmTBI. Our findings contribute to the larger body of literature examining the utility of biomarkers for chronic TBI and underscore the importance of examining heterogeneity within this population.

## CRediT authorship contribution statement

**Abigail B. Waters:** Writing – review & editing, Writing – original draft, Visualization, Methodology, Formal analysis, Data curation, Conceptualization. **Samantha H. Penhale:** Writing – review & editing, Formal analysis, Data curation. **Shoumi Sarkar:** Writing – review & editing, Validation, Methodology, Formal analysis, Conceptualization. **Somnath Datta:** Writing – review & editing, Validation, Supervision, Methodology, Formal analysis. **Damon G. Lamb:** Writing – review & editing, Software, Resources, Project administration. **Claudia Robertson:** Writing – review & editing, Supervision, Resources, Project administration, Methodology, Investigation, Funding acquisition. **Richard Rubenstein:** Writing – review & editing, Supervision, Resources, Project administration, Methodology, Investigation, Funding acquisition. **Amy K. Wagner:** Writing – review & editing, Supervision, Resources, Project administration, Methodology, Investigation, Funding acquisition. **Firas Kobeissy:** Writing – review & editing, Supervision, Resources, Project administration, Methodology, Investigation, Formal analysis, Data curation. **Kevin Wang:** Writing – review & editing, Supervision, Resources, Project administration, Methodology, Investigation, Funding acquisition, Formal analysis. **John B. Williamson:** Writing – review & editing, Supervision, Resources, Project administration, Methodology, Investigation, Funding acquisition, Conceptualization.

## Funding

This work was funded by Department of Defense grant W81XWH-19-2-0012. Any opinions, findings, conclusions or recommendations expressed in this material are those of the author(s) and do not necessarily reflect the views of the Department of Veteran's Affairs, the Department of Defense, or the United States Government.

## Declaration of Competing Interest

The authors declare the following financial interests/personal relationships which may be considered as potential competing interests: KKW is a shareholder of Gryphon Bio, Inc. a company interested in developing CNS therapeutics and diagnostics. The other authors have no other competing interests.

## Data Availability

De-identified clinical and behavioral data are publicly available in the Federal Interagency Traumatic Brain Injury Research Informatics System (FITBIR). Biomarker and imaging data are available by contacting the investigators.
