# Supplementary material for: Linked alterations in structure and autoimmunity biomarkers in remote mild-to-moderate TBI: A multi-modal brain imaging study
Source: Neuroimage Clin. 2025 Jun 30;47:103838. doi: 10.1016/j.nicl.2025.103838 (PMC12270732; doi:10.1016/j.nicl.2025.103838)
Supplement: Supplementary Data 1 [file mmc1.docx]

**Supplement 1. Relative weights of TBI Burden score derived from PCA**

| Supplemental Table 1. Relative weights of TBI Burden score derived from PCA (Component 1) | | | | | | | | | | | | |
| --- | --- | --- | --- | --- | --- | --- | --- | --- | --- | --- | --- | --- |
| **Component** | **1** | **2** | **3** | **4** | **5** | **6** | **7** | **8** | **9** | **10** | **11** | **12** |
| TBI |  |  |  |  |  |  |  |  |  |  |  |  |
| *1* | 0.34 | 0.31 | 0.16 | -0.41 | 0.07 | 0.13 | 0.06 | -0.29 | 0.27 | 0.32 | 0.39 | -0.09 |
| *2* | 0.41 | 0.10 | -0.26 | 0.12 | -0.34 | -0.02 | 0.32 | 0.02 | 0.13 | 0.21 | -0.27 | 0.16 |
| *3* | 0.36 | -0.22 | -0.09 | 0.28 | 0.43 | -0.10 | -0.06 | -0.30 | 0.26 | 0.17 | -0.13 | -0.05 |
| *4* | 0.19 | -0.41 | 0.16 | -0.15 | -0.10 | 0.17 | -0.12 | -0.10 | -0.08 | 0.22 | -0.61 | -0.27 |
| *5* | 0.14 | -0.35 | 0.18 | -0.15 | -0.22 | -0.13 | 0.04 | -0.17 | 0.34 | -0.26 | -0.03 | 0.21 |
| LOC |  |  |  |  |  |  |  |  |  |  |  |  |
| *1* | 0.15 | 0.24 | 0.77 | 0.24 | 0.10 | -0.33 | 0.26 | 0.21 | -0.13 | 0.06 | -0.07 | 0.04 |
| *2* | 0.17 | 0.20 | 0.20 | 0.52 | -0.40 | 0.39 | -0.48 | -0.23 | 0.02 | -0.11 | 0.00 | -0.06 |
| *3* | 0.11 | -0.11 | 0.04 | 0.13 | 0.23 | 0.69 | 0.53 | 0.15 | -0.07 | -0.29 | 0.18 | 0.03 |
| *4* | 0.11 | -0.21 | 0.07 | -0.07 | -0.03 | 0.22 | -0.32 | 0.64 | 0.15 | 0.42 | 0.11 | 0.38 |
| *5* | 0.04 | -0.10 | 0.07 | -0.04 | -0.06 | -0.02 | -0.09 | 0.39 | 0.34 | -0.13 | 0.10 | -0.77 |
| AOC |  |  |  |  |  |  |  |  |  |  |  |  |
| *1* | 0.34 | 0.34 | 0.03 | -0.49 | 0.19 | 0.15 | -0.31 | 0.07 | -0.17 | -0.35 | -0.32 | 0.06 |
| *2* | 0.39 | 0.17 | -0.36 | 0.06 | -0.32 | -0.25 | 0.18 | 0.26 | -0.18 | -0.13 | 0.20 | -0.11 |
| *3* | 0.36 | -0.16 | -0.15 | 0.25 | 0.45 | -0.19 | -0.22 | 0.11 | -0.13 | -0.17 | -0.10 | 0.03 |
| *4* | 0.17 | -0.35 | 0.10 | -0.14 | -0.14 | 0.00 | -0.10 | -0.13 | -0.69 | 0.19 | 0.35 | -0.15 |
| *5* | 0.14 | -0.32 | 0.18 | -0.15 | -0.21 | -0.14 | -0.03 | -0.02 | 0.12 | -0.45 | 0.24 | 0.23 |
| **Variance** | 0.51 | 0.66 | 0.75 | 0.82 | 0.88 | 0.91 | 0.93 | 0.96 | 0.97 | 0.99 | 0.99 | 1.00 |

**Supplement 2. Descriptions for each LICA-derived component.**

Component 1. Component was dominated by weak positive VBM (64%) effects widely distributed across the cortex.

Component 2. The bulk of variance was accounted for by FA (65%) and MD (27%), characterized by decreased FA in corpus callosum and increased MD in the ventricles (suggestive of increased ventricular size).

Component 3. The bulk of the variance was accounted for by MD (63%) and FA (24%), characterized by increased MD across the superior surface and reduced FA in the genu of the corpus callosum.

Component 4. The bulk of the variance was accounted for by MD (63%) and FA (33%), characterized by increased MD along the ventral surface of the frontal lobe and decreased FA in bilateral corticospinal tract and cerebral peduncle.

Component 5. Component was dominated by positive CT (76%) effects widely distributed across the cortex.

Component 6. The bulk of the variance was accounted for by FA (50%) and VBM (24%), characterized by increased FA in the corpus callosum and VBM in the cerebellum.

Component 7. The bulk of the variance was accounted for by MD (52%) and FA (39%), characterized by decreased MD and increased FA in posterior white matter tracts.

**Supplement 3. The impact of moderate TBI.**

When the analyses are repeated without the individuals who have moderate TBI, the strength of correlations between components and TBI burden are decreased from 0.02 – 0.08, as would be expected when reducing the spread of the TBI burden variable. Correlations between the LICA-derived components and the other variables (biomarkers and cognitive testing) were largely consistent (see below; correlation coefficients in red changed more than 0.10).

|  | Components | | | | | | |
| --- | --- | --- | --- | --- | --- | --- | --- |
|  | 1 | 2 | 3 | 4 | 5 | 6 | 7 |
| Biomarker |  |  |  |  |  |  |  |
| *GFAP* | 0.13 | 0.12 | -0.01 | 0.10 | -0.03 | -0.09 | -0.05 |
| *NFL* | 0.09 | 0.03 | 0.20 | -0.20 | -0.05 | -0.08 | 0.16 |
| *UCH-L1* | 0.03 | -0.02 | -0.06 | 0.42 | 0.08 | -0.08 | -0.02 |
| *Tau* | 0.11 | -0.05 | 0.09 | -0.05 | 0.01 | -0.17 | 0.22 |
| Cognitive |  |  |  |  |  |  |  |
| *WAIS-IV PSI* | 0.11 | -0.10 | 0.15 | -0.27 | 0.20 | 0.26 | -0.22 |
| *WAIS-IV WMI* | 0.27 | -0.05 | 0.03 | -0.14 | 0.22 | 0.29 | -0.21 |
